# Porcine model of progressive cardiac hypertrophy and fibrosis with secondary postcapillary pulmonary hypertension

**DOI:** 10.1186/s12967-017-1299-0

**Published:** 2017-10-06

**Authors:** Mariann Gyöngyösi, Noemi Pavo, Dominika Lukovic, Katrin Zlabinger, Andreas Spannbauer, Denise Traxler, Georg Goliasch, Ljubica Mandic, Jutta Bergler-Klein, Alfred Gugerell, Andras Jakab, Zsuzsanna Szankai, Levente Toth, Rita Garamvölgyi, Gerald Maurer, Frederic Jaisser, Faiez Zannad, Thomas Thum, Sándor Bátkai, Johannes Winkler

**Affiliations:** 1 Department of Cardiology, Medical University of Vienna, Währinger Gürtel 18-20, 1090 Vienna, Austria; 2 Institute of Diagnostic Imaging and Radiation Oncology, University of Kaposvar, Kaposvar, Hungary; 3 INSERM, UMRS 1138, Team 1, Centre de Recherche des Cordeliers, Pierre et Marie Curie University, Paris Descartes University, Paris, France; 4 Centre d’Investigation Clinique Inserm, CHU, Université de Lorraine, Nancy, France; 5 Institute for Molecular and Translational Therapeutic Strategies (IMTTS), Hannover, Germany

**Keywords:** Cardiac hypertrophy, PET-MRI imaging, Aortic isthmus stenosis, Translational large animal model, Gene expression

## Abstract

**Background:**

Meaningful translational large animal models for cardiac diseases are indispensable for studying disease mechanisms, development of novel therapeutic strategies, and evaluation of potential drugs.

**Methods:**

For induction of heart failure, cardiac hypertrophy and fibrosis, a bare metal stent was implanted in the descending aorta of growing pigs (n = 7), inducing pressure stress on the left ventricle (group HYPI). The constant stent size in growing pigs resulted in antegrade partial obstruction of the aortic flow with a gradual increase in afterload. Five pigs with sham intervention served as control. Serial haemodynamic, pressure–volume loop measurements and transthoracic echocardiography (TTE) were performed to detect developing pressure overload of the LV and cardiac MRI with late enhancement for measuring LV and RV mass and ejection fraction.

**Results:**

At 5-month follow-up, CT and contrast aortography, and intraluminal echocardiography confirmed aortic isthmus stenosis with a mean trans-stenotic gradient of 64 ± 13.9 mmHg. Invasive haemodynamic measurements revealed a secondary increase in pulmonary artery pressure (44.6 ± 5.1 vs 25.9 ± 6.2 mmHg, HYPI vs control, p < 0.05). TTE and ex vivo analyses confirmed severe concentric LV hypertrophy (mean circumferential wall thickness, 19.4 ± 3.1, n = 7 vs 11.4 ± 1.0 mm, n = 5, HYPI vs controls, p < 0.05). The LV and RV mass increased significantly, paralleled by increased isovolumic relaxation constant (tau). Histological analyses confirmed substantial fibrosis and myocyte hypertrophy in both LV and RV. Expressions of ANP, BNP, and miRNA-29a were up-regulated, while SERCA2a and miRNA-1 were down-regulated. Plasma NGAL levels increased gradually, while the elevation of NT-proBNP was detected only at the 5-month FUP.

**Conclusion:**

These data prove that percutaneous artificial aortic stenosis in pigs is useful for inducing clinically relevant progredient heart failure based on myocardial hypertrophy and fibrosis.

**Electronic supplementary material:**

The online version of this article (doi:10.1186/s12967-017-1299-0) contains supplementary material, which is available to authorized users.

## Background

Cardiac hypertrophy is an adaptive response to several cardiovascular disorders including volume- or pressure overload, cardiomyopathies, gene mutations, or loss of contractile mass after myocardial infarction [1]. Hypertrophic growth accompanies many heart diseases such as hypertension, ischemia, and valvular diseases. An increasing disease burden is attributed to patients with a phenotype cluster of adiposity, hyperlipidemia, and diabetes [2]. Persisting hypertrophy progresses to heart failure with its broad palette of clinical consequences such as intensive medical treatment, increased hospitalization and mortality or malignant arrhythmia.

Myocardial fibrosis is characterized by activation of fibroblasts to a myofibroblast phenotype, and diffuse interstitial collagen deposition. On the molecular level, myocardial fibrosis is the result of the imbalance of pro-fibrotic factors such as growth factors and hormones, which result in enhanced synthesis of cross-linked collagen types I and III, and a reduced collagen degradation by matrix metalloproteinases and anti-fibrotic factors, such as adipokines, or natriuretic peptides [3].

Blocking neurohumoral activation and relieving hemodynamic overload is the current standardized treatment of heart failure in the clinical practice. However, because of the molecular complexities of cardiac hypertrophy, fibrosis, and heart failure, new strategies are focusing on intervention on the intrinsic cardiac responses at the cardiomyocyte, interstitial and the intravascular level. In order to test new potential treatment regimens, adequate and meaningful animal models are necessary.

To mimic secondary cardiac hypertrophy, surgical interventions in rodents are frequently used [4]. A partial occlusion of the ascending or descending aorta by a ligature or clip (aortic banding) in rodents which is followed by an abrupt increase in pre-occlusion pressure is most commonly used to model left ventricular hypertrophy (LVH) caused by aortic stenosis [5, 6]. Other available technologies include genetic manipulation, gradual aortic constriction through banding, and pulmonary or renal artery constriction, recently reviewed in [7]. However, while mouse and rat models have allowed for important observations, their translational value is restricted because of anatomical and molecular divergence from humans. In contrast, several large animals and particularly pigs better resemble human anatomy and (patho)physiology and are therefore important for translational cardiovascular research.

Surgical aortic banding was shown to trigger cardiac hypertrophy in pigs [8], inducing abrupt pressure overload of the left ventricle (LV), which might be accompanied by acute increase in wall stress leading to acute heart failure [9]. Yarbrough et al. reported the induction of pressure overload in pigs by progressive cuff inflation of the ascending aorta over a period of 4 weeks [10]. While LV ejection fraction was preserved, LV mass was increased rapidly twofold. A less invasive approach to induce chronic LVH and heart failure with preserved ejection fraction (HFPEF) was reported by implantation of a 90-day-release subcutaneous depot of the aldosterone agonist deoxycorticosterone acetate (DOCA) in pigs together with a Western diet of salt, sugar, and fat [11]. Increased levels of blood cholesterol and triglycerides were found, and a moderate hypertrophy developed during the 3-month follow-up [11]. This method seems to be the most appropriate one for mimicking human metabolic syndrome and gradual development of hypertension and its haemodynamic consequences (e.g. diastolic dysfunction) to date. However, chronic administration of DOCA directly elevates renin-aldosterone system biomarkers, and therefore the measurement of standard heart failure markers may not be really informative.

We aimed to develop a large animal model of percutaneous artificial aortic isthmus stenosis induced by percutaneous implantation of peripheral bare metal stents (BMS) in descending aorta of juvenile pigs as the constant size of stent in growing pigs results in an antegrade partial obstruction of the aortic flow with gradual increase in afterload. With this method, we were able to successfully create chronic LVH, diastolic dysfunction and secondary pulmonary hypertension predicting HFPEF for use in translational research.

## Methods

### Study design

**Fig. 1:**
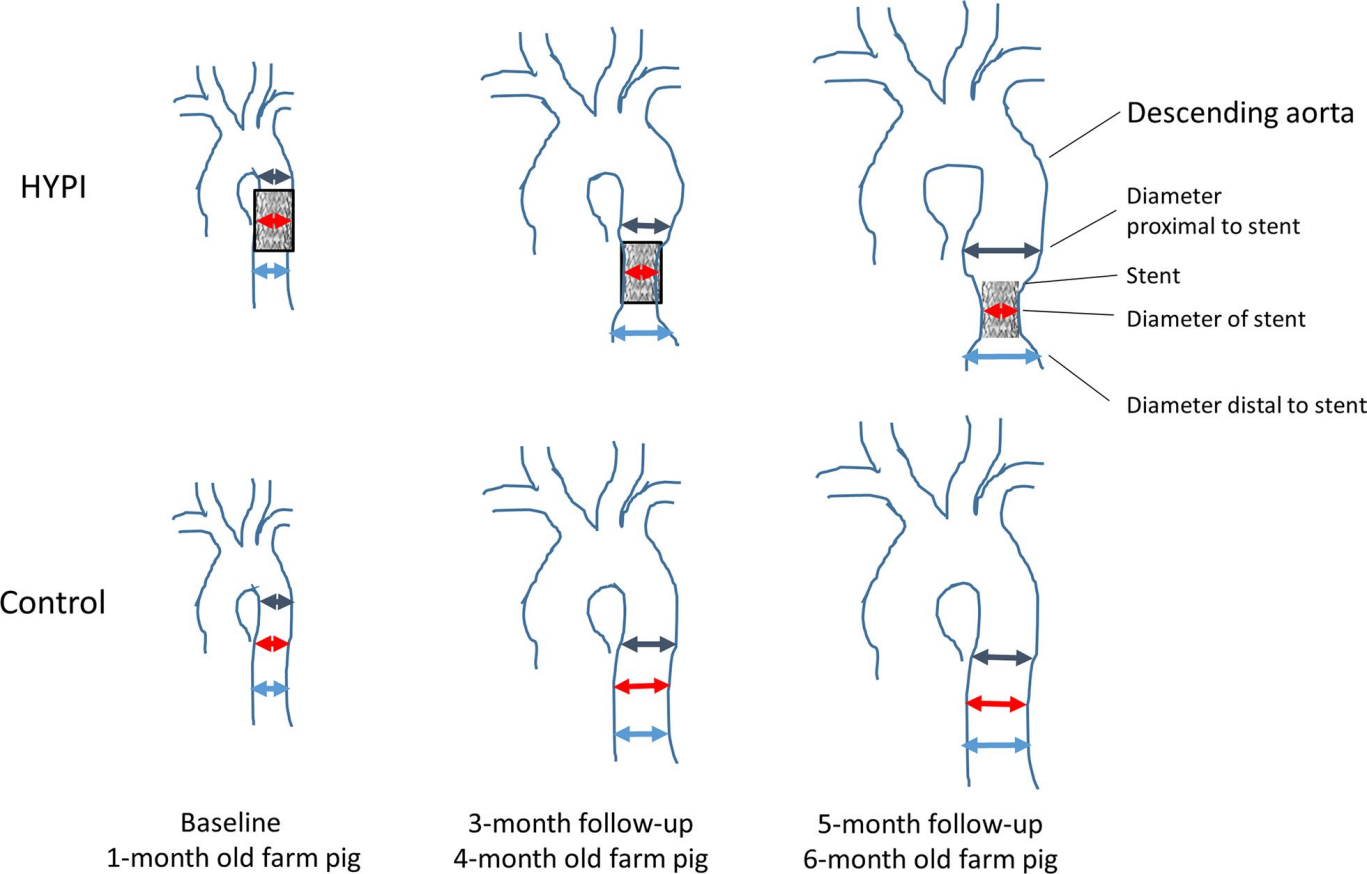
Schematic illustration of the development of artificial aortic isthmus stenosis. HYPI: group of cardiac hypertrophy. The implanted stent became ingrown in the aorta wall, and its constant size does not allow the normal growth (lateral and longitudinal) of the descending aorta at the site of the stenting. Note the pre- and post-stenotic dilation of the descending aorta adjacent to the stent, due to the turbulent flow

Figure 1 shows the study design. Based on our preliminary experiences with this model, a 5-month follow-up period was optimal for a gradual and steady development of left ventricular hypertrophy with simultaneous slow increase of the pulmonary arterial pressure. The study was terminated after 5 months due to increasing risk of LV hypertrophy complications, such as decompensation of the aortic stenosis inducing pulmonary congestion or lethal arrhythmias.

### Animal preparation

Domestic pigs (male, 15 kg, n = 12) were fasted overnight and were sedated with 12 mg/kg ketamine hydrochloride, 0.04 mg/kg atropine and 1.0 mg/kg xylazine, followed by intratracheal intubation. The anesthesia was then continued with 1.5–2.5 vol% isofluran, 1.6–1.8 vol% O_2_ and 0.5 vol% N_2_O. During anesthesia, continuous monitoring of the O_2_ saturation and ECG were performed. After surgical preparation of the right femoral artery and vein, 200 IU/kg unfractionated heparin was administered intravenously, and 6F introducers (Medtronic, Minneapolis, MN, USA) were placed in both vessels. In order to prevent acute or subacute stent thrombosis, dual oral antiplatelet medication (75 mg clopidogrel, 100 mg aspirin) was given for 1-month after stent implantation, followed by continuous treatment with 100 mg per os aspirin until the end of the experiment.

Transthoracic echocardiography (TTE) (GE, Chicago, IL, USA) was performed at baseline, and at the 2, 3, 4 and 5 month FUP to measure left ventricular (LV) diastolic wall thickness of the septum and posterior wall in M-mode and 4-chamber view, and to measure the diastolic function of the left ventricle by pulsatile wave Doppler, including A- and E-wave velocity and e′.

Right heart catheterization was performed via 5F pigtail (Cordis, a CardinalHealth Company, Dublin, OH, USA) catheter, and the pressures of the right atrium (RA) and ventricle (RV) and pulmonary artery (PA) were measured. The left heart cathetherization was performed with another 5F pigtail catheter (Cordis, a CardinalHealth Company, Dublin, OH, USA) measuring the pressures of the left ventricle (LV), ascending and descending aortae immediately after the arcus aortae (proximal to implanted stent) and at the diaphragma level (distal to implanted stent). Pressure–volume (PV) loop investigation was performed using the Powerlab Equipments and software, adapted for large animals (ADInstruments, Dunedin, New Zealand).

**Fig. 2:**
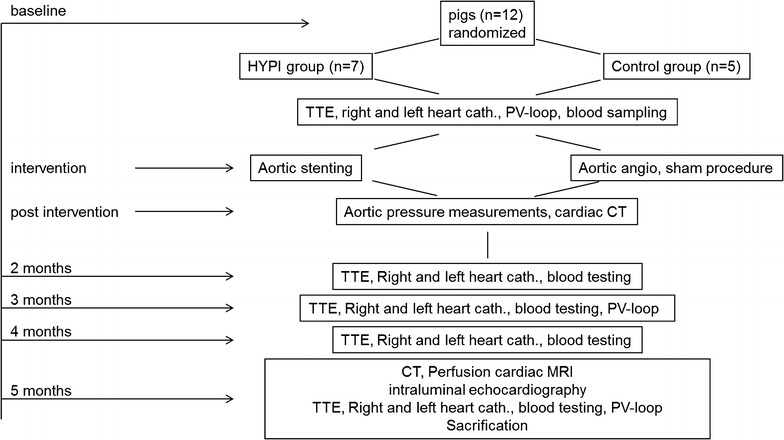
Study design. *HYPI* group of cardiac hypertrophy, *TTE* transthoracic echocardiography, *cath* catheterization, *CT* computed tomography, *PV-loop* pressure–volume loop measurements, *angio* angiography, *MRI* magnetic resonance imaging

After completing the baseline investigations, bare metal stents (9 mm of diameter, 20 mm of length, Cordis S.M.A.R.T. CONTROL, Cordis, Fremont, California) were implanted in the descending aorta immediately under the arcus aortae (Figs. 1 and 2) in 7 pigs (HYpertrophic PIgs, Group HYPI), while sham procedures were performed in 5 pigs (Group Control). Pressures of the ascending and descending aortae pre- and post-stent were measured via the pigtail catheter.

After the final MRI investigation at the 5-month follow-up, during continuous deep anaesthesia (1.5–2.5 vol% isofluran, 1.6–1.8 vol% O_2_ and 0.5 vol% N_2_O), the pigs were administered an additional dose of intravenous heparin (10,000 U). Euthanasia was performed by giving 10 mL intravenous saturated potassium chloride (10%), in accordance with accepted guidelines.

### Follow-up investigations

Serial TTE and invasive ascending and descending aorta pre- and post-stent, LV, RV, RA and PA pressures were measured at 2, 3, 4 and 5-month FUP. PV loop measurements were performed at 3 and 5 month time points. Diameters of the descending aorta were measured at the site of the stent, pre-stent (proximal to stent) and post-stent (distal to stent) in HYPI animals, and at the corresponding places in controls. Mild oversizing of the stent was chosen to avoid slipping of the stent to a distal location.

Additionally, aortography by computer tomography, intraluminal echocardiography (ILE) and magnetic resonance imaging with late enhancement (MRI + LE) were performed to measure stenosis grade, LV and RV systolic function and myocardial fibrosis at the end of the experiments. The final CT and MRI + LE was performed within 1 day of angiography and final invasive measurements, followed by immediate scarification of the animals.

Descriptions of the imaging modalities are detailed in the Additional file 1.

The animals were sacrificed 5 months after stent implantation, and the hearts were explanted. Myocardial tissue and plasma samples were collected for further analyses. Myocardial tissue samples from the LV, RV, RA and left atrium (LA) were immediately transferred to either RNAlater solution (Qiagen, Germany) or 7.5% formalin. For histological sections, tissues were embedded in paraffin and cut to 4–5 µm slices. For quantification of collagen and fibrosis, the samples were stained with HE, PicroSirius Red and MOVAT pentachrome and microscopy photographs were taken on an Olympus microscope IX83. For histological analyses, samples with similar orientation and with interstitial collagen staining were selected. Fibrosis, i.e. collagen quantity, was assessed by computerized planimetry by using ImageJ using thresholding of stained images. Myocyte cross-sectional areas were calculated with Olympus CellSens Dimension software by manually defining cell borders, using an average of at least forty cells per sample.

The animal studies were approved by the local Ethical Committee of the Experimental Animal Care Committee of the University of Kaposvar, Hungary.

### Gene expression analyses

For RNA isolation, tissue samples of around 25 mg were cut to small pieces, transferred to 700 µL Qiazol solution and homogenized using a Precellys system (Peqlab, Germany). Total RNA including small RNA was extracted using the miRNeasy Mini Kit (Qiagen, Germany) on a Qiacube according to the manufacturer’s instruction. RNA quantities were assessed on a Nanodrop 1000 (Thermo Fisher). RNA quality was checked on RNA Nano chips on the Agilent 2100 Bioanalyzer (Agilent Technologies). All samples resulted in RIN values over 7 and intact 18S and 28S bands.

For mRNA quantification, RNA was transcribed to cDNA using the Qiagen QuantiTect Kit according to the manufacturer’s instructions and qPCR was performed using the Qiagen qPCR Sybr Green Kit with gene specific primers (ANP forward: CAGGAGGGGGAAACCAGAAAG, reverse: CAGCAAATTCTTGAAATCCATCAGG, BNP forward: CGCAGTAGCATCTTCCAAGTC, reverse: ACCTCCTGAGCACATTGCAG, Serca2a forward: TTTCGCTCGAGTTGAACCTT, reverse: GAGCAGGAGCATCATTCACA, beta-Actin forward: TCAACACCCAGCCATGTAC, reverse: CTCCGGAGTCCATCACGATG) on an Applied Biosystems 7500 Fast qPCR system (Thermo Fisher). The mean of two technical replicates was used for each individual sample. A standard curve was prepared and run for each gene for assessing PCR efficiency. Expression quantities are expressed relative to beta-Actin.

Sequences of pig miRNAs were derived from the miRBase database [12]. For miRNA quantification small RNAs were transcribed to cDNA using the Qiagen miScript II RT Kit according to the manufacturer’s instructions and qPCR was performed using the Qiagen qPCR Sybr Green Kit with the Universal Primer and an miRNA specific primer (mir-1: GCAGTCTGGAATGTAAAGAAG, mir-29a: CGGACCTAGCACCATCTGAA, let7a: GCAGTGAGGTAGTAGGTTGT, miR-26: GCACGGTTCAAGTAATCCAGG, miR-27: GCTAGTTCACAGTGGCTAAG, Qiagen miScript primer assay for RNU6B). The mean of two technical replicates was used for each individual sample. Expression rates were corrected for PCR efficiencies using dilution curves and the expressions of the candidate reference genes (let7a, miR-26, miR-27, RNU6B) were entered in Normfinder [13] for identification of the most stable transcripts. Accordingly, miRNA expression was analyzed in relation to the geometric mean of miR-27 and let7a.

### Statistical analyses

Statistical significance was calculated by testing HYPI (n = 7) vs control animals (n = 5) using one-way ANOVA with repeated measurements, testing the between-group differences. In case for which the difference was significant, we supplemented the statistical comparison with two-sided t-tests. For statistical comparison of the groups, the parameters were assigned to the following imaging clusters: TTE, invasive pressure measurements, cardiac MRI, PV loop, histology, circulating biomarker and qPCR. Interdependent parameters determined from the same curve or figure (e.g. maximal and minimal values of the same measure or one parameter is a derivate of another parameter) were accounted as one measurement. The levels of statistical significance were determined after Bonferroni correction for the following imaging groups: invasive pressure measurements (p < 0.005), cardiac MRI (p < 0.01), PV loop measurements (p < 0.01) and qPCR (p < 0.01). For TTE, histology and circulating biomarker comparisons (≤ 5 parameters were compared) p < 0.05 was considered as statistically significant. All analyses were performed by an independent observer, who was unaware of the treatment groups.

## Results

### Imaging of experimental aortic isthmus stenosis

**Fig. 3:**
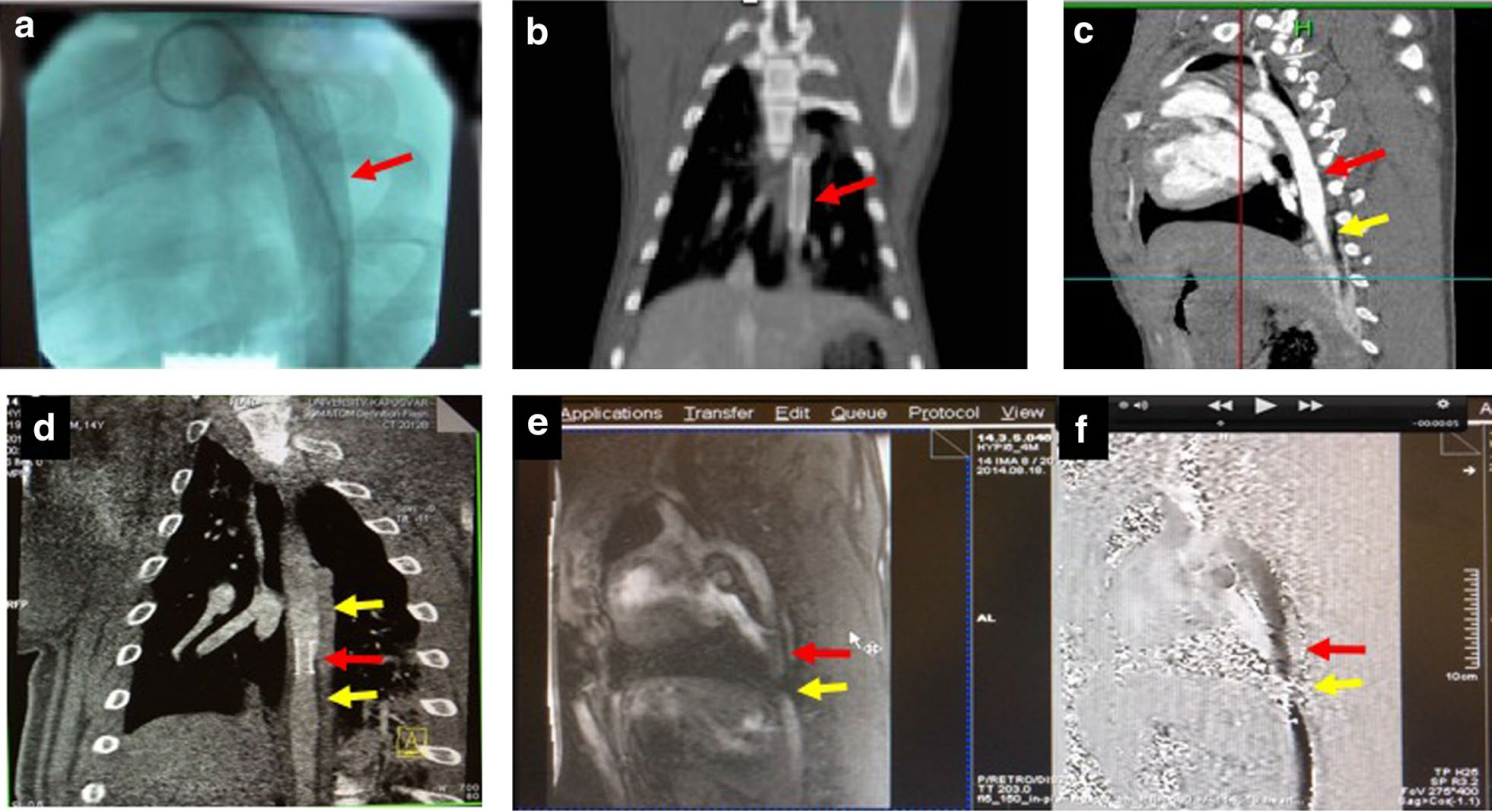
Creation of artificial aortic isthmus stenosis and myocardial hypertrophy in pigs via percutaneous stent implantation in the descending aorta. **a** Contrast angiography in a farm pig (15 kg) immediately after implantation of a bare metal stent (0.9 mm diameter, 20 mm length). **b** Computed tomographic (CT) aortography shows the location of the stent in anteroposterior view immediately after implantation. **c** Contrast-enhanced CT (lateral view) after stent implantation. **d** Magnetic resonance imaging (MRI) 5 months after stenting of the descending aorta displays the stent (red arrow), the pre- and poststenotic dilation (yellow arrows). Anteroposterior view. **e** Contrast enhanced MRI at 5-month follow-up shows the severely limited flow (yellow arrow) through the stent (red arrow). Lateral view. **f** Systolic frame from phase-contrast MRI cines; Aorta thoracalis, diameter: 15 mm, stent diameter: 9 mm, aliasing signal-fast moving blood in the stent area. (Maximum velocity 300 m/s equivalent with 56 mmHg pressure gradient on trough–plane images); 5-month follow-up

**Fig. 4:**
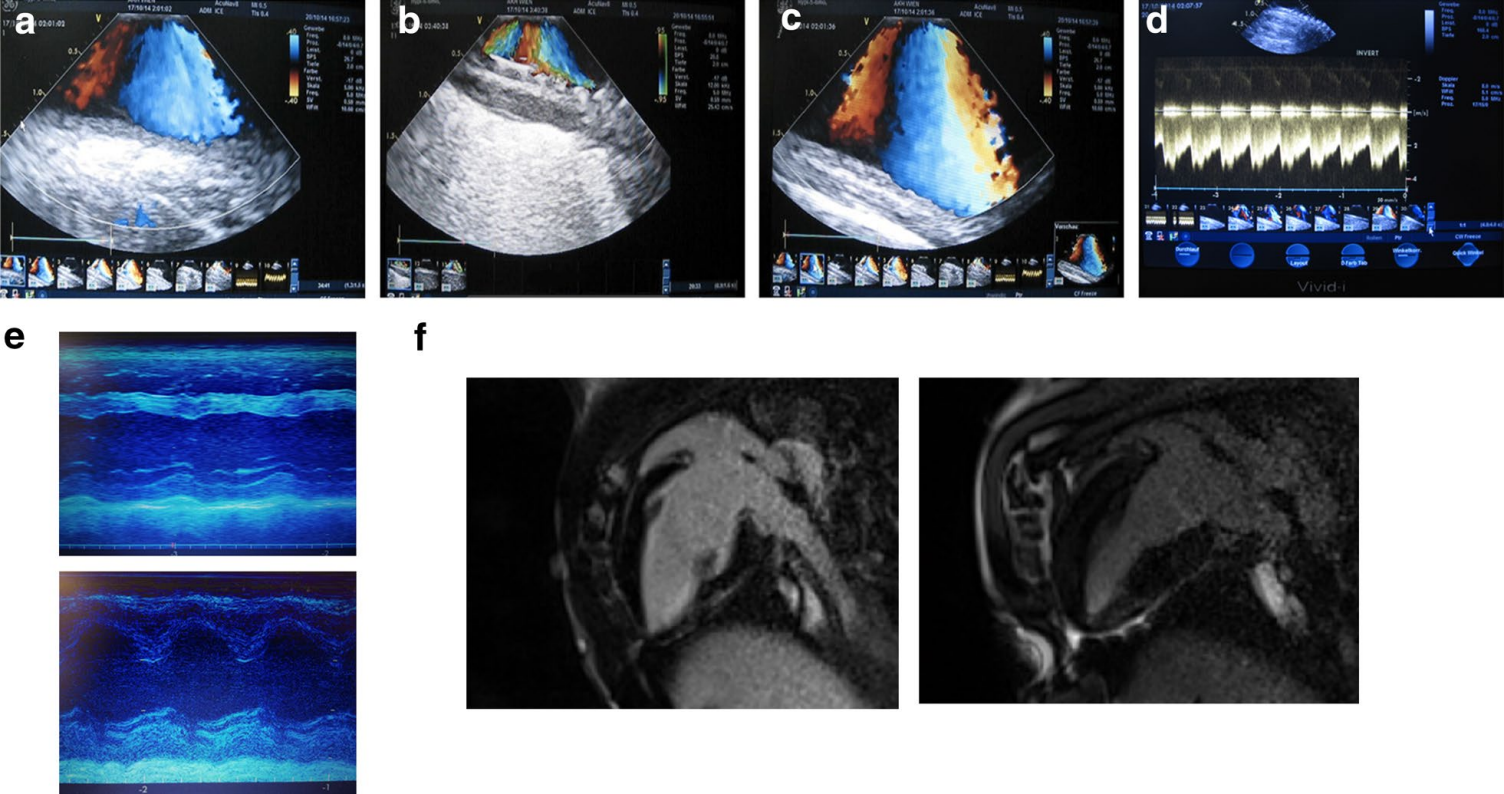
Imaging of aortic isthmus stenosis and development of myocardial hypertrophy. **a**−**d** Intraluminal echocardiography proximal to stent (**a**), in stent (**b**) and distal to stent (**c**) in the descending aorta 5 months after percutaneous stent implantation showing narrowing of the aorta in the stent level with tissue proliferation between the stent and the growing aorta in juvenile pigs (**b**). **d** Pulsatile Doppler measurements of the transstenotic gradient. **e** M-mode echocardiography displays concentric hypertrophy 5 month after creation of artificial aortic isthmus stenosis (lower panel) as compared to normal (upper panel). f Long axis cardiac magnetic resonance imaging confirms the severe concentric hypertrophy in HYPI pigs (right panel) in contrast with normal left ventricular myocardium of a control pig (left panel)

The diameters of the descending aorta are listed in Table 1. Figure 3a–c display the stent in the descending aorta immediately after implantation by contrast aortography and computed tomography in the juvenile pigs. At 5-month follow-up, images of the descending aorta showed the stent with in-stent and distal persistent stenosis in each case confirmed by computed tomography, with severely compromised flow through the stenosis confirmed by perfusion MRI angiography (Fig. 3d–f). ILE of the descending aorta displayed the stent, and the persistent connective tissue hyperplasia with narrowed lumen of the aorta at the stent level, accompanied by prestenotic dilation of the aorta and turbulent flow within the stent (Fig. 4a–c). Continuous wave Doppler revealed a mean transstenotic gradient of 55 ± 6 mmHg (Fig. 4d). TTE (Fig. 4e) and cardiac MRI (Fig. 4f) revealed thickened myocardium, with developing diastolic dysfunction at the 5-month follow-up.

**Table 1:** Diameter of the descending aorta measured by angiography at monthly intervals

Time		HYPI (n = 7)		Co (n = 5)		p value
		mean	SD	mean	SD	
Baseline						
Mid Ao desc		8.46	0.33	8.38	0.26	0.674
Prox Ao desc		8.64	0.46	8.50	0.16	0.523
Dist Ao desc		8.51	0.44	8.44	0.17	0.730
1 month						
Mid Ao desc	Stent*	9.06	0.08	10.28	0.68	0.001
Prox Ao desc		12.44	0.68	12.22	0.76	0.606
Dist Ao desc		11.70	1.27	11.50	0.88	0.768
2 months						
Mid Ao desc	Stent*	9.07	0.08	12.32	0.86	0.000
Prox Ao desc		14.11	0.61	13.60	0.79	0.232
Dist Ao desc		13.39	0.68	12.72	0.72	0.132
3 months						
Mid Ao desc	Stent*	9.11	0.09	15.00	1.12	0.000
Prox Ao desc		16.26	0.94	15.52	0.91	0.205
Dist Ao desc		15.01	0.64	14.02	1.06	0.070
4 months						
Mid Ao desc	Stent*	9.14	0.13	16.72	1.11	0.000
Prox Ao desc		19.21	0.65	18.02	1.28	0.058
Dist Ao desc		16.36	0.98	15.54	0.97	0.183
5 months						
Mid Ao desc	Stent*	9.17	0.15	18.29	1.34	0.000
Prox Ao desc		22.14	1.11	20.61	0.85	0.028
Dist Ao desc		18.46	0.80	16.77	0.67	0.003

The implanted 9 mm diameter stent was slightly oversized in order to prevent the moving of the stent to distal. The constant size of the stent in the growing aorta resulted in an artificial stenosis at the mid part of the descending aorta with pre- and post-stenotic dilation

*Prox* proximal, *Dist* distal, Stent* diameter of the mid descending aorta at the place of the stent

### Serial TTE data of the LV hypertrophy

Serial TTE revealed developing severe concentric LVH with an end-diastolic septum and posterior wall thickness of 19.6 ± 2.2 and 15.8 ± 1.3 mm (mean circumferential 19.4 ± 3.1 mm, n = 7), respectively, in the HYPI group at the final follow-up, in contrast with the normal wall thickness in controls (11.4 ± 1.0, n = 5). The final E/A ratio was 0.92 ± 0.56, E/e′ ratio 9.2 ± 1.5 (Table 2, Fig. 4e), indicating diastolic dysfunction in the HYPI group. No correlation was found between the E/e` ratio and the LV end-diastolic pressure, similar to data reported earlier by Previtali et al. [14].

**Table 2:** Serial measurements of the left and right ventricular systolic and diastolic parameters

Parameter	Baseline		2 months FUP		3 months FUP		4 months FUP		5 months FUP	
	HYPI (n = 7)	Control (n = 5)	HYPI (n = 7)	Control (n = 5)	HYPI (n = 7)	Control (n = 5)	HYPI (n = 7)	Control (n = 5)	HYPI (n = 7)	Control (n = 5)
Weight (kg)	15 ± 3	15 ± 1	34 ± 4	34 ± 3	53 ± 8	53 ± 5	74 ± 8	74 ± 4	93 ± 9	94 ± 7
TTE										
LA diameter (mm)	26 ± 2	25 ± 2	29 ± 3	28 ± 2	31 ± 2	29 ± 2	42 ± 2*	30 ± 2	45 ± 3*	34 ± 3
E/A ratio	1.2 ± 0.2	1.3 ± 0.3	1.1 ± 0.1	1.2 ± 0.2	1.0 ± 0.1	1.2 ± 0.3	0.9 ± 0.1*	1.2 ± 0.1	0.9 ± 0.6*	1.2 ± 0.2
E/e′ ratio	5.4 ± 0.2	4.8 ± 2.1	6.3 ± 0.8	5.3 ± 0.7	6.5 ± 1.1	5.2 ± 1.1	8.8 ± 0.5*	4.9 ± 0.6	9.2 ± 1.5*	5.4 ± 2.2
*Mean circumferential end-diastolic wall thickness (mm)*	*11.1* ± *0.9*	*11.1* ± *0.8*	*12.7* ± *1.9*	*11.2* ± *1.1*	*13.0* ± *1.2*	*10.9* ± *0.9*	16.6 ± 1.7*	*9.8* ± *1.1*	19.4 ± 3.1*	*11.4* ± *1.0*
Invasive pressure measurements (pigtail)										
Heart rate (bpm)	93 ± 29	98 ± 29	93 ± 8	93 ± 12	110 ± 22	92 ± 18	90 ± 11	87 ± 4	110 ± 19*	90 ± 20
LV syst. pressure (mmHg)	112 ± 16	104 ± 20	140 ± 19	111 ± 11	151 ± 11*	110 ± 17	160 ± 6*	126 ± 6	167 ± 15*	116 ± 23
LV end-diastolic pressure (mmHg)	8 ± 8	7 ± 7	20 ± 6	8 ± 10	22 ± 10*	17 ± 4	22 ± 2*	7 ± 2	23 ± 14*	13 ± 5
LV dP/dT (mmHg/s)	979 ± 150	1010 ± 329	1403 ± 431	1105 ± 370	1604 ± 388*	1061 ± 511	1597 ± 95*	1095 ± 75	1610 ± 416*	1186 ± 403
Aorta asc. syst. pressure (mmHg)	110 ± 12	105 ± 23	134 ± 18	109 ± 28	141 ± 14*	100 ± 23	150 ± 6*	123 ± 5	155 ± 18*	108 ± 13
Aorta asc. diast. pressure (mmHg)	72 ± 9	67 ± 15	78 ± 9	67 ± 13	79 ± 10	63 ± 11	94 ± 5	76 ± 4	95 ± 20*	70 ± 8
Post-stent aorta desc. syst. Pressure (mmHg)	108 ± 13	108 ± 15	93 ± 6	108 ± 9	87 ± 13	110 ± 10	89 ± 2	119 ± 6	93 ± 14*	107 ± 10
Post-stent aorta dessc. diast. pressure (mmHg)	69 ± 13	71 ± 16	57 ± 13	61 ± 9	57 ± 11	73 ± 6	64 ± 3	75 ± 6	74 ± 15*	69 ± 9
Aorta transstenotic gradient (mmHg)	2 ± 2	2 ± 1	40 ± 15*	7 ± 14	51 ± 20*	4 ± 4	61 ± 7*	4 ± 3	64 ± 14*	1 ± 4
RA pressure (mmHg) A valve	6 ± 6	5 ± 5	10 ± 7	10 ± 9	10 ± 7	10 ± 6	11 ± 2	6 ± 3	12 ± 9*	8 ± 5
RA pressure (mmHg) V valve	5 ± 4	5 ± 5	10 ± 5	9 ± 4	16 ± 3	9 ± 6	11 ± 2	5 ± 2	12 ± 4*	7 ± 5
RV syst. pressure (mmHg)	20 ± 8	18 ± 6	29 ± 11	21 ± 5	42 ± 7*	28 ± 4	47 ± 4*	23 ± 5	51 ± 5*	30 ± 4
RV end-diast. Pressure (mmHg)	9 ± 4	10 ± 4	10 ± 2	9 ± 1	8 ± 4	5 ± 5	12 ± 2	5 ± 2	13 ± 5*	9 ± 4
RV dP/dT (mmHg/min)	160 ± 43	172 ± 93	297 ± 108	175 ± 63	402 ± 40*	162 ± 58	425 ± 35	237 ± 37	473 ± 153*	273 ± 195
Pulmonary artery systolic pressure (mmHg)	24 ± 6	25 ± 6	29 ± 12	21 ± 8	35 ± 15	25 ± 4	43 ± 4	22 ± 3	45 ± 5*	26 ± 6
Pulmonary artery diastolic pressure (mmHg)	11 ± 1	12 ± 4	17 ± 8	10 ± 3	22 ± 10*	13 ± 2	25 ± 4*	11 ± 1	27 ± 6*	10 ± 2
Pulmonary artery mean pressure (mmHg)	21 ± 2	18 ± 4	23 ± 10	16 ± 5	29 ± 12	19 ± 3	34 ± 4*	17 ± 2	36 ± 5*	18 ± 4

Data were collected using transthoracal echocardiography (TTE), invasive pressure measurements (pigtail) in hypertrophic pigs (HYPI) and in controls

*Italics* Parameters characterizing LV hypertrophy

*LV* left ventricular, *RA* right atrial, *RV* right ventricular

* p < 0.05 for TTE and p < 0.005 for invasive pressure measurements parameter (ANOVA after Bonferroni correction) between the groups at the same time point

### Serial invasive pressure measurements of experimental myocardial hypertrophy

**Fig. 5:**
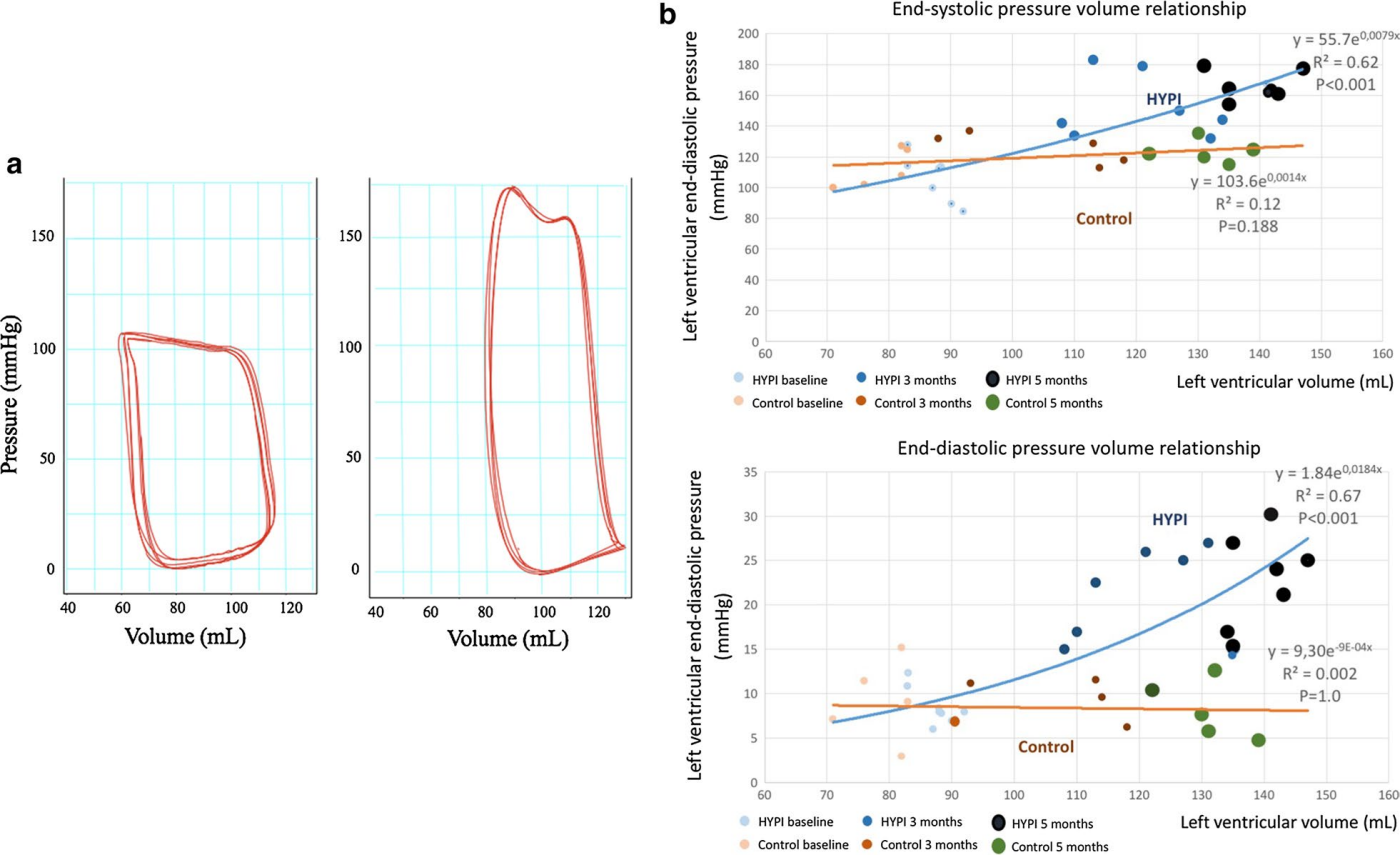
**a** Typical pattern of the myocardial hypertrophy in pressure-loop measurement indicating increased systolic pressure and decrease in stroke volume at the 5-month follow-up in normal (left) and HYPI (right) pigs. **b** End-systolic (ESPVR) (upper panel) and end-diastolic pressure–volume relationship (EDPVR) (bottom) showing clear leftward shift of the curves

The PV loops showed typical features of LVH with increased peak systolic pressure and decrease in stroke volume (Fig. 5).

Control contrast angiography of the descending aorta showed slip of the stent to a position more distal (to the diaphragmal level) in 2 animals (stent diameter of 9 mm proved still to be too small), which, however, did not mitigate the LVH development.

Serial left and right heart catheterization revealed increasing transstenotic gradient with parallel increase in LV systolic, and end-diastolic pressure, as well as significant increase in pressures in the right heart. The secondary increase in systolic and diastolic right ventricular pressure, and pulmonary pressure reached significance at the 5-month follow-up (Fig. 5, Table 2).

Serial data of the PV-loop measurements are shown in Table 3. According to the increase in systolic and end-diastolic pressures of the LV, the end-systolic (ESPVR) and end-diastolic pressure volume relationship (EDPVR) curves showed leftward shift of the curves (Fig. 5).

**Table 3:** Serial measurements of the left and right ventricular systolic and diastolic parameters

	Baseline		3 months FUP		5 months FUP	
	HYPI (n = 7)	Control (n = 5)	HYPI (n = 7)	Control (n = 5)	HYPI (n = 7)	Control (n = 5)
Invasive pressure–volume loop measurements						
End-diastolic volume (mL)	87 ± 3	81 ± 6	127 ± 13	109 ± 25	133 ± 12	125 ± 10
End-systolic volume (mL)	38 ± 2	32 ± 7	54 ± 6	44 ± 12	53 ± 11	44 ± 4
Stroke volume (mL)	49 ± 5	49 ± 4	73 ± 8	65 ± 21	78 ± 9	81 ± 14
LV ejection fraction (%)	56 ± 4	60 ± 7	58 ± 3	59 ± 9	59 ± 4	65 ± 5
Stroke work (mmHg*mL)	4497 ± 145	4492 ± 1182	8692 ± 1017*	4813 ± 858	9407 ± 1027*	5335 ± 741
Cardiac output (L/min)	4.0 ± 0.2	4.4 ± 0.4	5.4 ± 0.8	5.2 ± 0.8	5.8 ± 0.8	5.8 ± 0.8
LV maximal pressure (mmHg)	105 ± 15	118 ± 14	150 ± 11*	124 ± 15	169 ± 18*	120 ± 5
LV minimum pressure (mmHg)	5 ± 2	2 ± 5	13 ± 3*	6 ± 2	14 ± 3*	6 ± 2
LV maximum dP/dt (mmHg/s)	1106 ± 164	1203 ± 155	1693 ± 679	1436 ± 136	1883 ± 384	1594 ± 276
LV minimum dP/dt (mmHg/s)	−1492 ± 246	−1485 ± 433	−1741 ± 126	−1620 ± 228	−1911 ± 480	−1616 ± 231
Mean arterial pressure (mmHg)	57 ± 9	60 ± 22	82 ± 7*	61 ± 4	91 ± 11*	68 ± 3
*LV maximal dV/dt (mL/s)*	*428* ± *135*	*428* ± *58*	*693* ± *94*	*592* ± *186*	527 ± 89*	*425* ± *57*
Arterial elastance (mmHg/mL)	2.4 ± 0.4	2.5 ± 0.2	3.0 ± 0.9	2.5 ± 0.3	3.2 ± 0.7*	2.4 ± 0.2
Tau (ms)	23.8 ± 6.7	25.7 ± 5.8	32.5 ± 1.9	26.8 ± 3.3	39.1 ± 8.2*	24.9 ± 4.7
MRI at 5 months						
LV end-diastolic volume (mL)					128 ± 16	109 ± 12
LV end-systolic volume (mL)					52 ± 10	38 ± 8
LV stroke volume (mL)					76 ± 12	72 ± 10
LV ejection fraction (%)					59 ± 6	66 ± 6
*LV mass (g)*					*151 ± 27*	*95 ± 17*
RV end-diastolic volume (mL)					97 ± 13*	78 ± 5
RV end-systolic volume (mL)					53 ± 15	37 ± 4
RV ejection fraction (%)					47 ± 10	52 ± 6
*RV mass (g)*					*82 ± 9*	*57 ± 6*
LV peak filling rate (mL/s)					349 ± 114*	514 ± 81

Data were collected using invasive pressure measurements with pressure–volume loop catheter in hypertrophic pigs (HYPI) and in controls

*Italics* Parameters characterizing LV hypertrophy

*LV* left ventricular, *RA* right atrial, *RV* right ventricular

* p < 0.01 for pressure–volume loop and MRI parameter (ANOVA after Bonferroni correction) between the groups at the same time point

### cMRI + LE results

Five-month cMRI + LE showed preserved LV ejection fraction (EF), with a trend to a decrease in right ventricular EF. In parallel with the significant increase in LV mass, the LV peak filling rate decreased in the HYPI animals, confirming the TTE findings. This model might also be considered as a model of heart failure with preserved EF (HFPEF).

### Obduction and histology

**Fig. 6:**
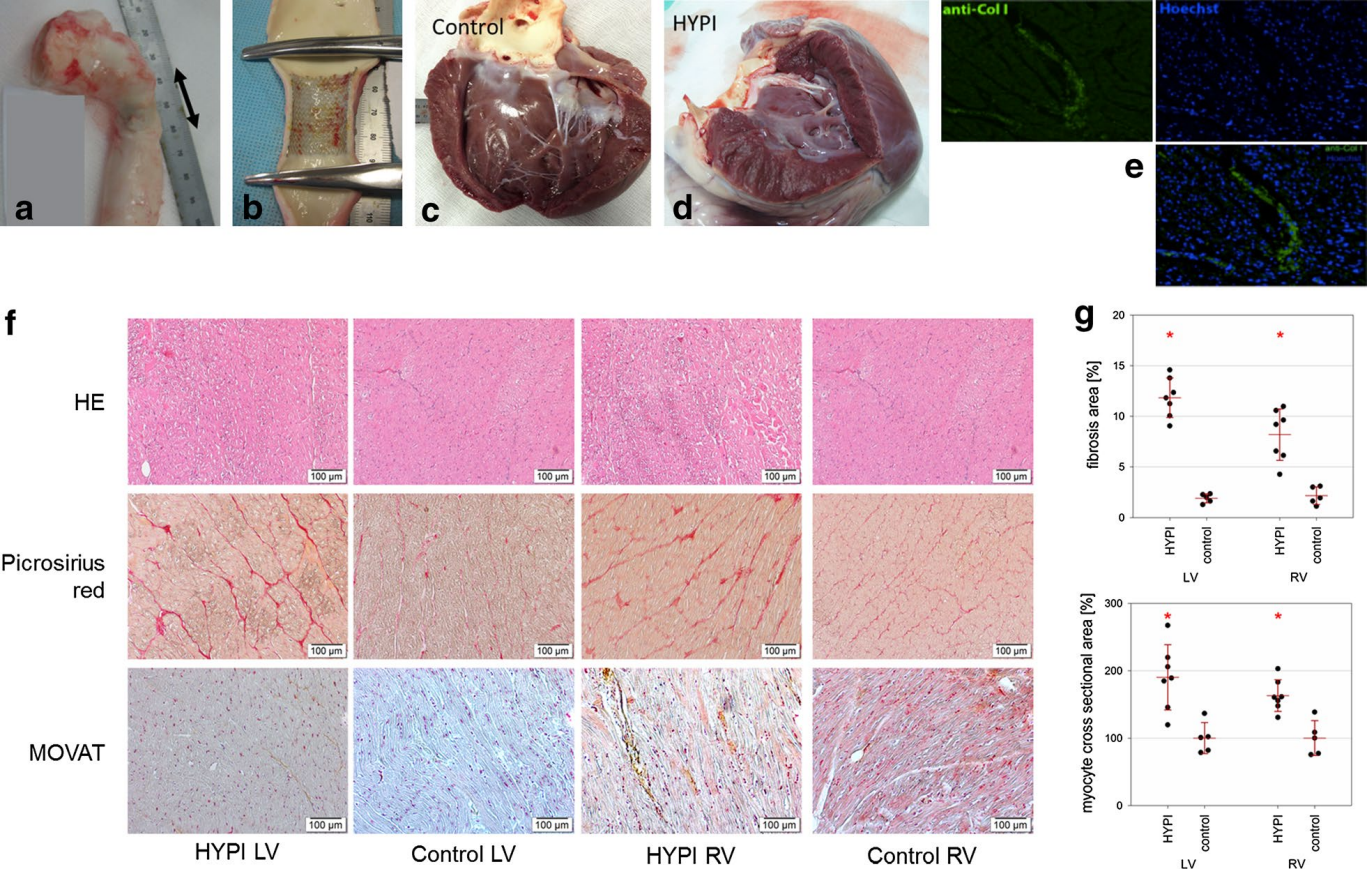
Macroscopic and microscopic imaging of the aortic isthmus stenosis, myocardial hypertrophy and fibrosis. **a** Explanted descending aorta shows pre- and poststenotic dilation of the corresponding aorta segments 5-month after stent implantation (arrow). **b** Longitudinal cut of the aorta displays the stent in the aorta. **c** and **d** Left ventricular myocardium in a control animal and in a hypertrophic pig (HYPI), showing severe concentric hypertrophy in the HYPI animal. **e** Anti-collagen staining of the hypertrophied myocardium shows collagen accumulation as a proof of myocardial fibrosis. (left: anti-collagen staining, right: DAPI counterstaining, bottom: merged). f Histologic images of the left ventricle (LV) and right ventricle (RV) of hypertrophic (HYPI) and control animals. HE, Picrosirius red and MOVAT staining display pronounced interstitial fibrosis in the HYPI animals both in the LV and RV. **g** Quantification of fibrosis of the myocardial samples: significantly higher amount of fibrosis in the HYPI animals. Mean ± SD, *p < 0.05 (ANOVA) between the control (n = 5) and HYPI groups (n = 7)

After the 5-month follow-up, the obduction revealed macroscopic constriction of the aortic part with the stent with pre- and post-stenotic dilation of the aorta (Fig. 6a, b). The slowly developing aortic isthmus stenosis with consequent rise in pre-stent systolic blood pressure was caused by the increasing mismatch of the stent and the increasing aorta diameter in the growing animals. The stent intimal hyperplasia was negligible. One stent was pulled more proximal during the extension of the aorta by the normal growing body and broke at the proximal part (Additional file 1: Figure S1) without any clinical signs or aortic wall rupture. Macroscopic severe concentric myocardial hypertrophy was found in every animals in the HYPI group, compared to the normal wall thickness of controls (Fig. 6c, d). The lungs of the HYPI animals showed mild macroscopic congestion, evidenced by reddish coloration of the lung tissue (not shown).

Histological preparations and staining of the LV myocardial tissue with PicroSirius red and an anti-collagen antibody showed a significant increase of collagen deposition as a characteristic feature of tissue fibrosis (Fig. 6e–g). The degree of the LV and RV fibrosis were significantly (p < 0.05) higher with 11.8 ± 2.0 and 8.2 ± 2.5% in the HYPI and 1.9 ± 0.4 and 2.2 ± 0.9% in the control group for LV and RV, respectively. The LV cardiomyocyte cross-sectional areas were higher in both the HYPI LV and the HYPI RV compared to control groups (1002 ± 255 µm^2^ vs 527 ± 122 µm^2^ for LV and 627 ± 91 µm^2^ vs 385 ± 100 µm^2^ for RV; p < 0.05).

### Circulating biomarkers of cardiac fibrosis

**Fig. 7:**
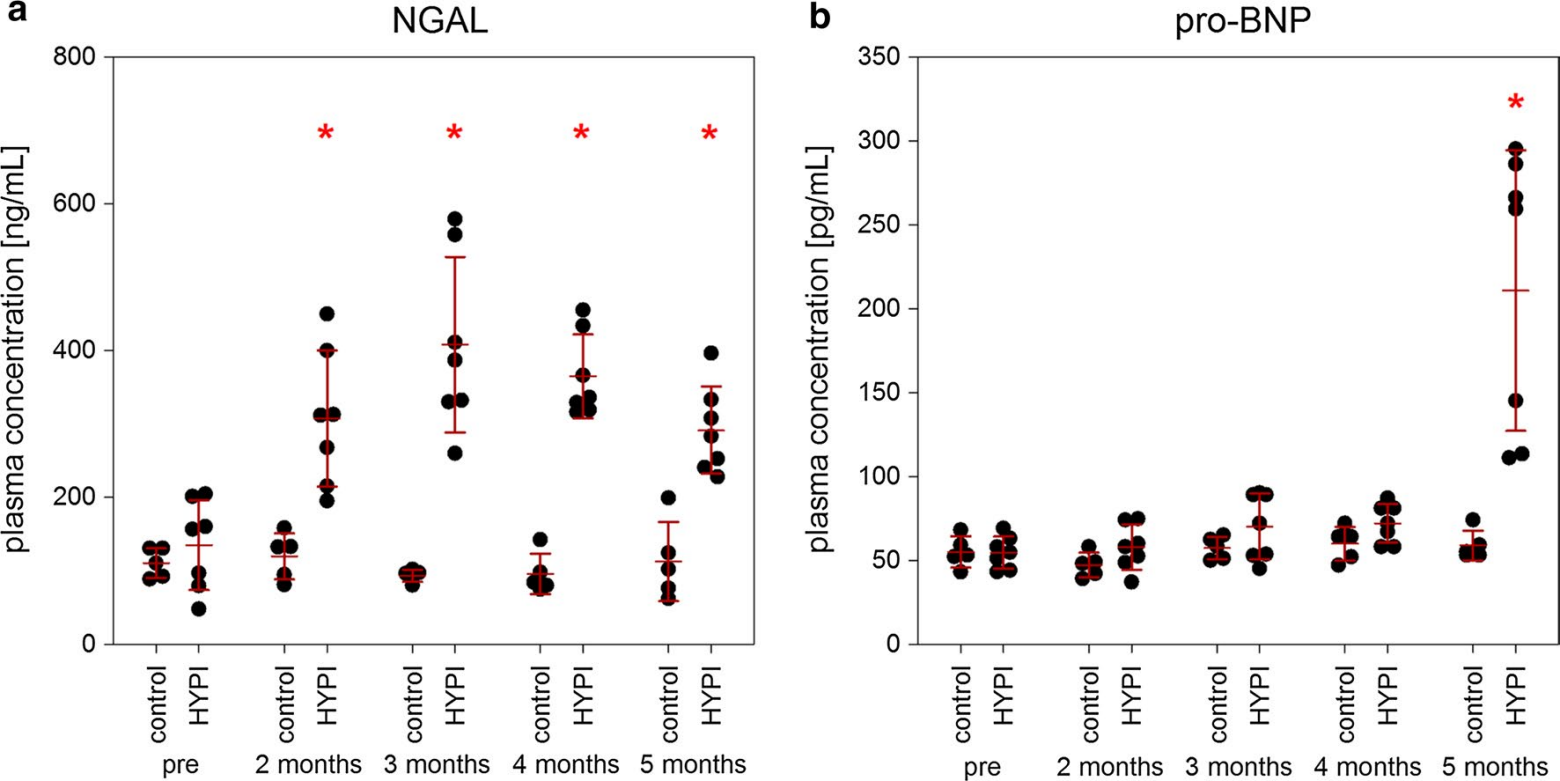
Serial measurements of plasma NGAL and proBNP levels. **a** Rapid increase in NGAL was detected already 2 months after creation of artificial aortic isthmus stenosis, even when the transstenotic gradient was not yet significant. **b** Late elevation of proBNP in the plasma was observed, indicating a compensatory mechanism of aortic stenosis and developing heart failure. Results are expressed as mean ± SD, *p < 0.05 (ANOVA) between the groups at the same time points (n = 7, HYPI, n = 5, control)

Plasma NGAL increased significantly after 2 months, when the myocardial hypertrophy was clinically not yet pronounced, reached a plateau at 3 months (Fig. 7a). In contrast the plasma proBNP increased only at the 5 months follow-up (Fig. 7b).

### Molecular markers of cardiac hypertrophy and fibrosis

**Fig. 8:**
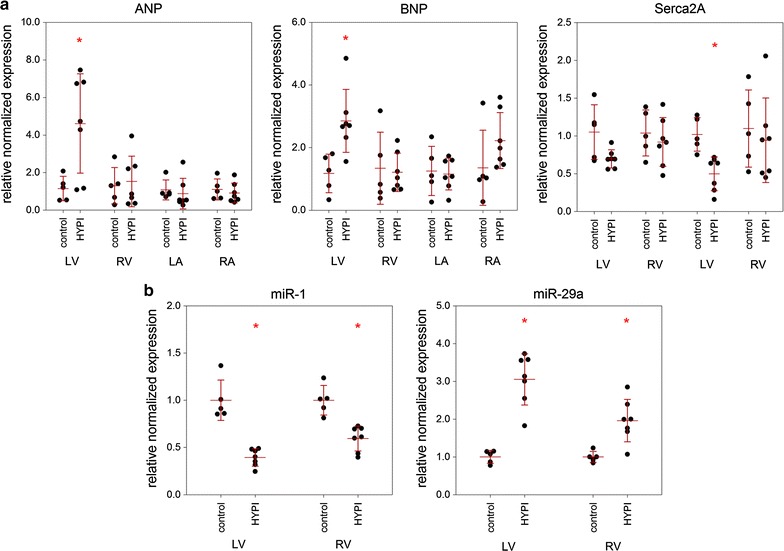
Molecular proof of cardiac hypertrophy and fibrosis, by myocardial gene expression of atrial natriuretic peptide (ANP), brain natriuretic peptide (BNP) and SERCA2a and microRNA-1 and 29 expression in left (LV) and right ventricle (RV) of pigs with cardiac hypertrophy compared to controls. **a** Gene expression in LV, RV, left atrium (LA), and right atrium (RA) of pigs with cardiac hypertrophy (HYPI) induced by aortic isthmus stenosis 5 months after stent implantation. ANP and BNP are up regulated in the LV, and the calcium ATPase SERCA2a showed diminished expression in the LA. **b** Significant alteration of the miRNA expression pattern was detected, including down regulation of miR-1 and upregulation of miR-29a in both ventricles. Results are expressed as mean ± SD, *p < 0.05 (ANOVA) between the groups (n = 7, HYPI, n = 5, control)

To confirm the changes associated with cardiac hypertrophy on the molecular level, we examined the expression of genes known to be up or down regulated in response to hypertrophy. As expected, the natriuretic peptide ANP and BNP, which are only marginally expressed in adult ventricular tissue under normal circumstances, show enhanced expression levels in the LV tissue in hypertrophic pigs. LV ANP expression was increased more than threefold, and BNP levels more than twofold compared to the values of the control animals (Fig. 8a). In contrast, the mRNA levels were not significantly altered in the LA, RA and RV (Fig. 8a). SERCA2a expression was decreased to 71% in the LV and to 48% in the LA (Fig. 8a).

We also examined the expression of selected microRNAs, which have been described to play a role in cardiac hypertrophy and fibrosis in animal models and in the human pathologic condition. We found depletion of the cardiomyocyte growth-repressing miR-1 to 50–66% in both the LV and RV of pigs with cardiac hypertrophy (Fig. 8b). In addition, miR-29a expression was increased 2- to 3-fold in the RV and LV, respectively.

## Discussion

For the development of novel and better treatment options for cardiac hypertrophy and fibrosis, relevant and reliable translational animal models are necessary. Here for the first time we report a percutaneous method to create slowly developing pressure overload of the LV causing pre-stenotic systolic hypertension, myocardial hypertrophy with LV diastolic dysfunction and secondary pulmonary hypertension in a relevant translational large animal model. The internally placed stent, in-grown in the aortic wall, precluded the normal extension of the aorta during the normal body growth. Comprehensive serial invasive hemodynamic measurements with serial TTE and ILE imaging allowed detailed monitoring of the gradual development of cardiac hypertrophy and fibrosis, confirmed by cardiac MRI and CT. Together with macroscopic and histological observations, these data show that the model is closely related to the condition of human heart failure and myocardial fibrosis by hypertension and aortic stenosis. Important new insights gained from this study are the animal model of progressing heart failure, with development of postcapillary pulmonary hypertension, and high translational value for the transition of HFPEF to HFREF.

Recognizing the necessity of large animals for modeling human cardiac diseases, surgical aortic banding of the aorta has been performed in pigs, inducing abrupt changes in the hemodynamics, causing acute heart failure [9, 15] or by implanting subcutaneous DOCA-depots [11] combined with Western diet. In contrast to these methods, our procedure is the first minimal invasive percutaneous intervention method, mimicking the gradual development of aortic stenosis paralleled by severe LV hypertrophy, diastolic dysfunction and secondary (postcapillary) pulmonary hypertension. In contrast with previous reports with shorter monitoring times post-aortic banding, the longer follow-up (5-month) and steady progression of the hemodynamic changes due to artificial aortic stenosis corresponds more closely to human conditions. The longer follow-up period also allowed us to the development of post-capillary pulmonary hypertension, which has not been described previously. The elevated pressures in the right heart due to LV diastolic dysfunction are equivalent to HFPEF. Up to now, by either aortic banding or a DOCA infusion pump, no data were reported on pressure changes of the right heart. Using serial measurements of the left and right heart haemodynamics with pig tail catheter and LV pressure–volume loop measurements, we confirmed the slowly developing increase in pre-stenotic systolic pressure with a trans-stenotic gradient up to mean 64 mmHg, accompanied by significantly increased stroke work and severe concentric LV wall thickening (circumferential wall thickness of mean 19 mm) confirmed by TTE, cardiac MRI and finally with ex vivo investigations. The gradual increase in transstenotic gradient and severe concentric LV hypertrophy induced diastolic dysfunction, evidenced by decreased E/A ratio, increased E/e′ ratio by TTE, end-diastolic pressure of the LV by LV haemodynamics and arterial elastance and tau by pressure–volume loop measurements. Parallel with these changes, the plasma NGAL level was gradually increased, while a significant plasma proBNP level increase was observed only at the 5 month follow-up, indicating that NGAL level might be a more sensitive early stage heart failure marker.

The end-diastolic volume increased in both groups, due to the regular growth of the juvenile pigs, but a trend towards LV enlargement was observed in the HYPI animals as compared to controls, with a trend to lower LV and RV EF predicting the development of heart failure with reduced ejection fraction (HFREF), if longer follow-up had been done [16]. The transition of HFPEF to HFREF has no clear time or disease condition-related cut-off, and this process depends on continuous pressure or volume-overload and accompanying diseases in humans. Hence, our model with gradually increasing pressure overload in the circulation models this on-going detrimental progress on the heart well.

Right heart catheterization revealed a gradual increase in the RA, RV and pulmonary artery pressures, indicating development of post-capillary pulmonary hypertension, even if measurements of capillary wedge pressure were not performed. Since the animals had no primary lung diseases at the obduction, and that the pulmonary hypertension was developed in parallel with the aortic stenosis severity, we can exclude a primary pulmonary hypertension. In contrast with the DOCA-treated animals in the experiments of Schwarzl et al. [11], who classified their model as heart failure stage B (ACC/AHA guidelines), we observed mild lung congestion of the HYPI animals and an elevated pro-BNP level, which correspond with the HF stage of C at the final 5-month FUP [17].

According to the echocardiographic circumferential wall thickness measurements and the invasive pressure measurements, it seems that persistent increase in blood pressure for a longer time is necessary for manifest LV wall thickening; however, a definitive timely correlation between increase in systolic blood pressure and myocardial hypertrophy requires a histological verification, necessitating the harvesting of the animals at different time-points.

Histology and the changes in expression of cardiac mRNAs and miRNAs confirmed the regulation of important biomarkers for hypertrophy and fibrosis. The left ventricular expression of natriuretic peptides ANP and BNP was increased, confirming the cardiac origin of the elevated plasma pro-BNP levels. The ventricular expression of natriuretic peptides is a well-documented result of pressure- and volume stress [18, 19] and one of the hallmarks of congestive heart failure. The endocrine effects of ANP and BNP at least partially attenuate pressure- and volume stress. The cardiac sarco/endoplasmic reticulum Ca^2+^-ATPase SERCA2a is instrumental in maintaining calcium homeostasis and decreased expression and function aggravates heart failure [5, 20, 21]. SERCA2a dysregulation is one of the key features of heart failure of cardiomyocyte dysfunction in both experimental animal models and human patients [22], and downregulation was verified in the left atrium and left ventricle. These changes confirm that the expected molecular pathways for cardiac hypertrophy are stimulated in our pig model.

Similarly, several microRNAs that are known to play a role in hypertrophy are up or down regulated. Increased expression of miR-29a is consistently found in hypertrophic animal models and in human heart failure and also with cardiac hypertrophy and fibrosis in hypertrophic cardiomyopathy patients [23], highlighting their use as potential biomarker. The microRNA-1 is well documented to be decreased in heart failure and fibrosis as well as animal models of cardiac hypertrophy, similar to our model [24, 25].

### Limitation

We did not perform MRI at baseline in order to avoid exposing the small, 15 kg animals to more stress; MRI would have required either an additional anesthesia, or a longer anesthesia for transport of the intubated animals from MRI to the angiographic lab.

We have measured the dP/dt both with the pigtail catheter and also with the piezo transducer of the pressure–volume loop catheter. We are aware that the pig-tail catheter may underestimate the dP/dt; however, in contrast to the pig-tail catheter, the pressure–volume loop measurements are very rarely used in humans, and we strived for optimal translational value of our experiments. Additionally, the pressures of the pigtail catheter were calibrated before each measurement. The 15 kg pigs were 1 month old, in “child age” for pigs, with somewhat lower normal systemic blood pressure, therefore the dP/dt values (derivate of systolic pressure and time) around 1000 mmHg/s measured by the pig tail catheter may be considered as being within the normal range.

As the pig heart lies behind the sternum, the usual echocardiographic measurements are difficult. The measurements of the left atrium diameter were possible from parasternal short axis view, and the Doppler parameters from the apical 4-chamber views, while measurement of the other parameters were not possible for all pigs for all time points; therefore, we have not reported these parameters.

### Translational value of artificial percutaneous aortic stenosis for human diseases

Induction of gradual development of LV hypertrophy by minimally invasive percutaneous approach in our large animals successfully simulates human conditions with natural progressive sclerotizing aortic stenosis being hemodynamically compensated and asymptomatic for a long time. This model is in a good agreement with pathophysiologic concepts of myocardial hypertrophy with consequent cardiac fibrosis, hence LV dilation and postcapillary pulmonary hypertension on the molecular and physiological level, offering several molecular targets for potential antifibrotic treatment, or preventive medical approaches against complications of aortic stenosis. Our model is mainly designed for pressure overload induced cardiac hypertrophy and progressing heart failure. However, we believe it can be used as a general model to study the cellular and molecular mechanisms of cardiac fibrosis, which shares at least some common features regardless of the underlying cause (LV adverse remodeling after myocardial infarction, cardiomyopathy or others).

## Conclusions

Percutaneous implantation of BMSs in the descending aorta of juvenile pigs causing slowly developing artificial aorta isthmus stenosis successfully simulates cardiac hypertrophy and fibrosis and is in good agreement with pathophysiologic concepts of human disease on the molecular and physiological level. It is thus a useful model for researching molecular mechanisms of hypertrophy and for evaluation of potential treatments.

## Additional file

**Additional file 1.** Detailed description of pressure-volume loops measurements, Transthoracic intraluminal echocardiography, CT, and MRI; photographs of stents after explantation.

## Abbreviations

ANP: atrial natriuretic peptide
BNP: brain natriuretic peptide
CT-1: cardiotrophin-1
DOCA: deoxycorticosterone acetate
EDPVR: end diastolic pressure–volume relationship
EF: ejection fraction
ESPVR: end systolic pressure–volume relationship
Gal-3: galectin-3
HFPEF: heart failure with preserved ejection fraction
HFREF: heart failure with reduced ejection fraction
HYPI: hypertrophic pigs
ILE: intraluminal echocardiography
LE: late enhancement
LOX: lysyl oxidase
LV: left ventricle
LVH: left ventricular hypertrophy
MRI: magnetic resonance imaging
NOX: NAPDH oxidase
PET: positron emission tomography
PA: pulmonary artery
PV: pressure volume
RA: right atrium
RV: right ventricle
TTE: transthoracic echocardiography
