# Supplementary material for: Porcine model of progressive cardiac hypertrophy and fibrosis with secondary postcapillary pulmonary hypertension
Source: J Transl Med. 2017 Oct 6;15:202. doi: 10.1186/s12967-017-1299-0 (PMC5639584; doi:10.1186/s12967-017-1299-0)
Supplement: Supplementary file 1 — Additional file 1. Detailed description of pressure-volume loops measurements, Transthoracic intraluminal echocardiography, CT, and MRI; photographs of stents after explantation. [file 12967_2017_1299_MOESM1_ESM.pdf]

Porcine model of progressive cardiac hypertrophy and fibrosis with secondary postcapillary pulmonary hypertension

Mariann Gyöngyösi, Noemi Pavo, Dominika Lukovic, Katrin Zlabinger, Andreas Spannbauer, Denise Traxler, Georg Goliasch, Ljubica Mandic, Jutta Bergler-Klein, Alfred Gugerell, Andras Jakab, Zsuzsanna Szankai, Levente Toth, Rita Garamvölgyi, Gerald Maurer, Frederic Jaisser, Faiez Zannad, Thomas Thum, Sándor Bátkai, Johannes Winkler

Additional file 1

Methods

Pressure-volume loop measurements

The pressure-volume (PV) loops were constructed using a 5F multisegment PV catheter with pigtail end, an effective length of 122 cm, containing 12 electrodes and dual field configuration (ADInstruments, Oxford, UK). First, the catheter was calibrated by using the Volume Calibration Cuvette (ADInstruments, Oxford, UK) to convert volume units into mL. The PV pigtail catheter was connected to the MPVS-300 Pressure-Volume Unit (ADInstruments, Oxford, UK) via the CEC-PV Extension Cable (Venti-Cath to MPVS Ultra 10ft). After ex-vivo calibration according to the manufacturers LabChart software (ADInstruments, Oxford, UK), using the large-mammal workflow, the catheter was introduced into the LV and the recording was started. After reaching the steady-state pressures and volumes, 10 cycles of the heart beat were recorded. Alpha calibration was done using stroke volume (SV) measured by cardiac CT as a reference value. The end-diastolic and end-systolic volumes (mL), left ventricular (LV) ejection fraction (%), stroke work (mmHg*mL), cardiac output (L/min), arterial elastance (mmHg/mL) and the isovolumic relaxation constant (tau). The PV loop investigations were repeated at baseline, and at 3, and 5-month.

The volume of the LV was measured by cardiac computed tomography and the PV loops volumes were calibrated to these measurements.

Transthoracic echocardiography (TTE) and intraluminal echocardiography

TTE of the pigs was performed at baseline, and at the time points of the serial invasive investigations by using GE Vivid S6 Ultrasound machine (GE Healthcare, Chicaco, IL, USA). According to the anatomical location of the pig heart (laying behind the sternum), limited ultrasound positions could be achieved: parasternal short and long axis, and 4-chamber view, allowing the measurements of the wall thickness (mm) at the mid LV level, as well as measurements of the diastolic function parameters (E, A and e`waves).

Intraluminal echocardiography was performed by using ACUSON AcuNav V Ultrasound Catheter (Siemens, Malvern, PA, USA). The ultrasound probe was introduced into the arteria femoralis via 9F introducer, and pushed forwards to the aortic arch. Recording was performed in 2D mode, using also color and CW Doppler, to visualize the flow through the intraaortic stent and measure also the transstenotic gradient.

All echocardiographic and intraluminal ultrasound investigations were repeated at baseline, 2,3,4 and 5-month follow-up.

Contrast-enhanced computed tomography (CT) aortography

ECG-gated CT images were performed by using a 64-slice SOMATOM CT scanner (Siemens) with the following parameters: 172 mA, 130 kV, 128x0.625 mm, 1 sec rotation time, 8.0 sec can time, 490 mm scan length. The imaging was started with unenhanced scanning from the lung apex to the aorta iliaca bifurcation. Contrast-enhanced CT imaging were then performed after giving iodinated contrast material (iopamiro, total volume 80 mL) through a ear vein using an injector pump, and the images was repeated. The images were performed immediately after placement of the stent in the aorta descendent and at the 5-month follow-up.

For determination of the left ventricular volumes, the left ventricular analysis software (LVA at the Leonardo workstation, Siemens Medical Solutions, Erlangen, Germany) was used. The systolic and diastolic planes of the aortic and mitral valves and apex were marked manually. The epi- and endocardial contours were traced semiautomatically both in end-diastole and end-systole. The tracing was controlled for visual accuracy and adjusted manually, if necessary. The end-diastolic and end-systolic volumes were calculated automatically. These measurements were used for parallel volume calibration with the PV loop measurements.

Magnetic resonance imaging (MRI)

Cardiac and the aorta magnetic resonance imaging (MRI) were performed using 1.5 T Siemens Avanto Syngo B17 scanner (Erlangen, Germany) with a phased-array coil and a vector ECG system. Functional scans were acquired using a retrospective ECG-gated (HR: 80-100 beats/minute), steady-state free precession (SSFP - TRUFISP sequence) technique in short-axis and long-axis views using 1.2 ms echo time (TE), 40 ms repetition time (TR), 25 phases, 50º flip angle, 360 mm field-of-view, 8 mm slice thickness, and 256 x 256 image matrix. For perfusion images, intravenous bolus of 0.15 mmol/kg contrast medium was injected into the ear veinm and inversion-recovery sequences were detected.

Cardiac function was assessed from short axis cine-series (25 phases). Volumetric measurements of the left and right ventricle were performed using the freely available academic license software Segment version 1.9 (Medviso AB, Lund, Sweden), and the global left and right ventricular ejection fraction and left ventricular mass were calculated by measuring the end-diastolic (EDV) and end-systolic volumes (ESV) on short axis cine MRI images. Left ventricular peak filling rate was calculated from the calculated volume time curve data.

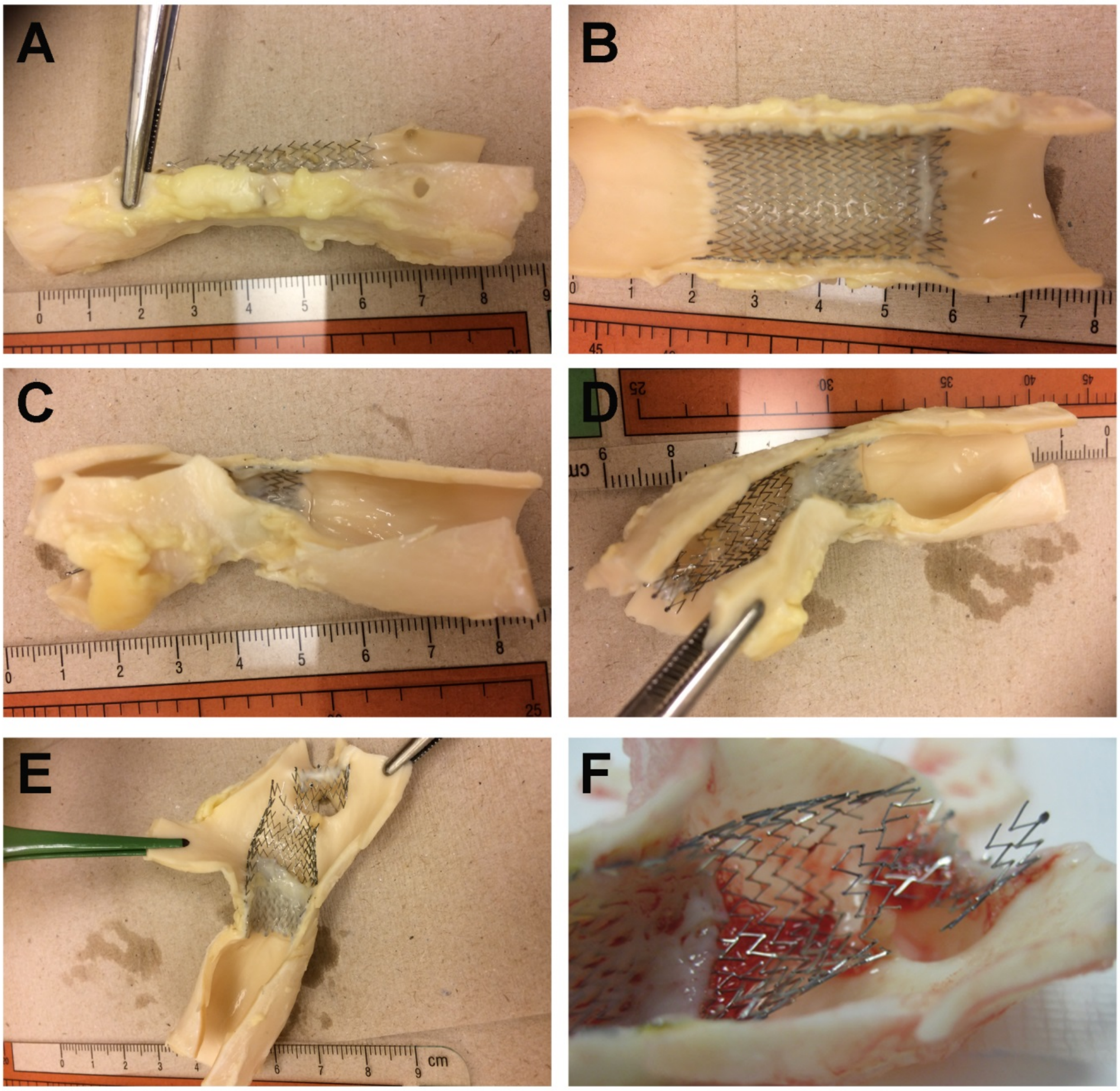

Additional File 1: Figure S1. Pictures of bare metal stents, which had been implanted in the aorta descendens directly below the arcus aortae in juvenile pigs, were taken after explantation. Panels **A** and **B**, **C** and **D**, and **E** and **F** each show the same stent, respectively. The stent in **E** and **F** was torn by prolongation of the aorta by the growing pig during the five months.
